# Supplementary material for: Forecasting China’s shipping indices based on modal decomposition and optimized deep learning integrated model
Source: PLoS One. 2025 Dec 8;20(12):e0336906. doi: 10.1371/journal.pone.0336906 (PMC12685171; doi:10.1371/journal.pone.0336906)
Supplement: Attachment — Submitted filename: response - Reviewer 2.pdf [file pone.0336906.s003.pdf]

Responses to the Reviewer 2's comments

We thank you for your careful review of our manuscript and many insightful comments. We have revised our manuscript according to your comments. Our point-by-point responses to your comments are given below, with your original comments copied in ***Italics*** for your convenience.

Comments:

1. *What is the difference between CPSO and PSO?*

   Response: The core distinction lies in the optimization mechanism: Standard PSO utilizes linear stochastic parameters for exploration, whereas Chaotic PSO (CPSO) incorporates nonlinear chaotic maps to dynamically adjust inertia weights or acceleration coefficients. This enables CPSO to escape local optima more effectively, enhance population diversity, and achieve superior convergence in complex, multimodal optimization problems, though it introduces marginal computational overhead due to chaotic dynamics calculations. In our study, CPSO's chaotic properties proved critical for optimizing BiLSTM hyperparameters in non-stationary shipping index forecasting.

2. *Need to add Justification for CPSO over PSO.*

   Response: We thank the reviewer for their valuable feedback. Our rationale for selecting CPSO over standard PSO lies in its ability to dynamically adjust search parameters through chaotic mapping, which effectively mitigates premature convergence and significantly enhances global exploration capability. This nonlinear chaotic mechanism not only increases population diversity, but also achieves superior and more stable solutions with faster convergence speed. In practical applications such as BiLSTM hyperparameter optimization, CPSO demonstrates stronger robustness and generalization ability, typically yielding optimized results with higher accuracy and reliability. The authors have included this justification below Table 6 in the revised manuscript.

3. *How long VMD, CPSO, BiLSTM takes (Time)?*

   Response: The computational time for each component in our VMD-CPSO-BiLSTM framework varies based on data characteristics and hardware configuration. In our experiments (conducted on Python 3.12 with an Intel i7-11700K processor and 32GB RAM), the approximate time costs are as follows:

1. VMD Decomposition: Requires 8-15 seconds per time series, primarily depending on the data length and the preset number of modes (K-value).

2. CPSO Optimization: Takes 45-120 seconds per run, influenced by population size, iteration count, and the complexity of chaotic mapping.

3. BiLSTM Training: Consumes 70-150 seconds per epoch, affected by network structure , sequence length, and batch size.

The integrated pipeline typically completes full training within 18-35 minutes.
